# Maternal obesity classes, preterm and post-term birth: a retrospective analysis of 479,864 births in England

**DOI:** 10.1186/s12884-019-2585-z

**Published:** 2019-11-21

**Authors:** Emma Slack, Kate E. Best, Judith Rankin, Nicola Heslehurst

**Affiliations:** Institute of Health & Society, Newcastle University, Baddiley-Clark Building, Richardson Road, Newcastle upon Tyne, NE2 4AX UK

**Keywords:** Pregnancy, Obesity, Preterm, Post-term

## Abstract

**Background:**

Preterm (< 37 weeks gestation) and post–term birth (≥42 weeks gestation) are associated with increased morbidity and mortality for mother and infant. Obesity (body mass index (BMI) ≥30 kg/m^2^) is increasing in women of reproductive age. Maternal obesity has been associated with adverse pregnancy outcomes including preterm and post–term birth. However, the effect sizes vary according to the subgroups of both maternal BMI and gestational age considered. The aim of this retrospective analysis was to determine the association between maternal obesity classes and gestational age at delivery.

**Methods:**

A secondary data analysis of 13 maternity units in England with information on 479,864 singleton live births between 1990 and 2007. BMI categories were: underweight (< 18.5 kg/m^2^), recommended weight (18.5–24.9 kg/m^2^), overweight (25.0–29.9 kg/m^2^) and obesity classes I (30.0–34.9 kg/m^2^), II (35.0–39.9 kg/m^2^), IIIa (40–49.9 kg/m^2^) and IIIb (≥50 kg/m^2^). Gestational age at delivery categories were: Gestational age at delivery (weeks): extreme preterm (20–27), very preterm (28–31), moderately preterm (32–36), early term (37, 38), full term (39–40), late term (41) and post–term (≥42). The adjusted odds of births in each gestational age category (compared to full-term birth), according to maternal BMI categories were estimated using multinomial logistic regression. Missing data were estimated using multiple imputation with chained equations.

**Results:**

There was a J-shaped association between the absolute risk of extreme, very and moderate preterm birth and BMI category, with the greatest effect size for extreme preterm. The absolute risk of post-term birth increased monotonically as BMI category increased. The largest effect sizes were observed for class IIIb obesity and extreme preterm birth (adjusted OR 2.80, 95% CI 1.31–5.98).

**Conclusion:**

Women with class IIIb obesity have the greatest risks for inadequate gestational age. Combining obesity classes does not accurately represent risks for many women as it overestimates the risk of all preterm and post-term categories for women with class I obesity, and underestimates the risk for women in all other obesity classes.

## Background

Preterm birth (< 37 weeks gestation) complications were a leading cause of death in children under 5 years, accounting for 1 million mortalities globally in 2015 [1]. Preterm babies are at increased risk of complications such as cerebral palsy, autism and disability, with the risk increasing with decreasing gestational age at birth [2]. Post–term birth (≥42 weeks gestation) is also associated with an increased risk of stillbirth, neonatal and infant death [3–5], and an increased risk of maternal morbidity due to fetal macrosomia [6], caesarean section [7–9], haemorrhage [8] and thromboembolic disease [7].

Obesity (body mass index (BMI) ≥30 kg/m^2^) in women of reproductive age is increasing. In the UK, 21.3% of women had an obese BMI in early pregnancy in 2016, and 28.4% had an overweight BMI (25.0–29.9 kg/m^2^) [10]. In the USA, 31.8% of women aged 20–39 had obesity in 2011–2012 [11]. The WHO divides obesity into sub-classes to reflect risk of co-morbidities: I (BMI 30.0–34.9 kg/m^2^), II (BMI 35.0–39.9 kg/m^2^) and III (BMI ≥ 40 kg/m^2^). Often obesity classes are not used in pregnancy guidelines or clinical practice. However, there is evidence to support their use, for example the odds of women developing gestational diabetes increases from 3.01 (95% CI 2.34–3.87) for obesity class I, to 5.55 (95% CI 4.27–7.21) for obesity class II/III [12]. Although class III obesity is the least prevalent obesity class, it is increasing at the most rapid rate over time, according to UK national data [13], and warrants further investigation. There is also an emerging interest in maternal extreme obesity (BMI ≥ 50 kg/m^2^) due to the increase in risks among this population [14, 15]. This presents an argument to further divide class III maternal obesity into IIIa (40–49.9 kg/m^2^) and IIIb (BMI ≥ 50 kg/m^2^).

Systematic reviews and meta-analyses show that maternal overweight and obesity are associated with preterm [16, 17] and post-term birth [18]. However, the effect sizes vary according to the subgroups of both maternal BMI and gestational age considered. For example, there is a lack of existing data on gestational age at birth and extreme obesity, and inconsistent definitions of gestational age categories used in published research [18] which do not reflect the sub-categorisations of term birth endorsed by the American College of Obstetricians and Gynecologists, the Society for Maternal-Fetal Medicine, and the Association of Women’s Health, Obstetric and Neonatal Nurses [19].

In light of the inconsistent use of definitions of both gestational age and obesity classes, and the lack of data on extreme obesity, this study aimed to determine the associations between gestational age categories and maternal obesity classes.

## Methods

This epidemiological study was a secondary analysis of an existing anonymised national dataset of routine maternity data NHS Trusts across England, UK [13]. The acquisition of the dataset has been described elsewhere [13, 20, 21] and included data on live births from 37 maternity units (24 NHS Trusts) and *n* = 738,307 births between 1989 and 2007. The dataset included booking information (i.e. first antenatal contact at approximately 12 weeks gestation) on measured maternal weight, measured maternal height, BMI calculated from measured height and weight, year, stage of pregnancy, maternal age, maternal ethnicity, maternal employment, parity, and Index of Multiple Deprivation (IMD) 2007 (a measure of area-level socioeconomic deprivation calculated from domains such as income, employment and health [22, 23], derived from mothers’ residential postcode at booking). Within the dataset, 13 NHS Trusts provided gestational age at delivery data and therefore this sub-sample of the original dataset was used in this secondary analysis. Although this dataset contains information on live births, no information is provided on viability.

The outcome was gestational age at delivery which was coded into seven categories: extreme preterm (20–27 weeks), very preterm (28–31 weeks), moderately preterm (32–36 weeks), early term (37–38 weeks), full term (39–40 weeks), late term (41 weeks) and post–term (≥42 weeks). (Term births were categorised according to the American College of Obstetrics and Gynaecologists [24].) Below the limit of viability, 24 weeks [25], the chance of infant survival is very low, but not impossible [26]. Therefore, we included births > 20 weeks gestational age. For extreme preterm birth, a sensitivity analysis was conducted, restricting to births ≥24 weeks gestation to explore the decision to include birth below the usual limit of viability (> 20 weeks).

The main explanatory variable was maternal BMI, which was coded into seven categories: underweight (< 18.5kgm^2^), recommended weight (18.5–24.9 kg/m^2^), overweight (25.0–29.9 kg/m^2^), obesity class I (BMI 30.0–34.9 kg/m^2^), class II (BMI 35.0–39.9 kg/m^2^), class IIIa (40–49.9 kg/m^2^) and class IIIb (BMI ≥ 50 kg/m^2^). Extreme obesity was defined as BMI ≥ 50 kg/m^2^ to be consistent with previous publications [13, 14, 27]. BMI < 11 kg/m^2^ and was recoded as missing as this is the lowest BMI for survival in women [28] and therefore it was assumed that any recorded BMI below this value was data entry error. BMI > 80 kg/m^2^ was recoded as missing according to previously used limits [14, 29]. Further analyses were carried out collapsing the four maternal obesity categories into one obesity category (BMI ≥30.0 kg/m^2^) to compare the findings with the analyses of maternal obesity classes I, II, IIIa and IIIb.

Additional socio-demographic explanatory variables were included in the adjusted analyses; variables chosen were hypothesised to be associated with both maternal obesity, and gestational age at delivery. These variables were: IMD (quintiles), ethnicity (White, South Asian, Black, Chinese/Other, Mixed ethnic group), employment (employed, not employed, home carer, higher education or education and/or age < 18), maternal age at booking (< 20, 20–24, 25–29, 30–34, 35–39, 40–44, ≥45 years), gestational age at booking (< 13, 13–25, ≥26 weeks) (please note that maternal age at booking and gestational age at booking were categorised due to non-linearity), parity (0 to 6) and region of England (East, London, North East, North West, South East, South West, West Midlands, Yorkshire and Humber) were analysed as categorical variables. Year of delivery was analysed as a continuous variable.

It was assumed the data were missing at random (where missingness can be explained by differences in observed data [30]). Therefore, multiple imputation using chained equations with 10 iterations was performed to impute missing data for BMI (17.9% missing), IMD (1.4%), ethnicity (15.0%), employment (27.6%), maternal age (0.7% missing), parity (1.7%) and gestational age at booking (0.4% missing) [31]. All of these variables were included as predictors in the chained equations along with year of delivery and region (both complete), and gestational age at delivery. Gestational age at delivery was missing in 0.4% of pregnancies, these data were not imputed as this is not the recommended approach for the outcome variable [31]. Births with missing gestational age were excluded from the analyses.

Univariable multinomial logistic regression was performed with gestational age at delivery category as the outcome variable and BMI category as the explanatory variable. The model estimated the odds of each category of gestational age delivery compared to full term according to each category of BMI compared to the recommended category. Multivariable multinomial logistic regression was similarly performed adjusting for employment, ethnicity, maternal age, parity, gestational age at booking and year of delivery. Models were performed using both original and imputed data.

## Results

The dataset included a total of 479,864 births between 1990 and 2007 of which 2954 (0.6%) were extreme preterm, 3815 (0.8%) were very preterm, 26,254 (5.5%) were moderately preterm, 81,448 (17.0%) were early term, 238,847 (49.8%) were full term, 93,237 (19.4%) were late term, 31,222 (6.5%) were post–term and 2087 (0.4%) had missing gestational age at delivery.

Following multiple imputation, 4.4% of women had an underweight BMI, 52.9% had a recommended BMI, 27.3% had an overweight BMI, 10.7% had class I obesity, 3.4% had class II obesity, 1.3% had class IIIa obesity, 0.1% had obese class IIIb obesity. Compared to the original dataset, in the imputed data there was a greater proportion of women in the categories of underweight (4.4% vs 3.3%), overweight (27.3% vs 26.2%) and obese class I (10.7% vs 9.8%), and a lower proportion of women in the categories of recommended weight (52.9% vs 55.6%), obese class II (3.4% vs 3.6%) and obese class IIIa (1.3% vs 1.5%). There was no difference for obese class IIIb (0.1% for original data and imputed data)). Demographic information for IMD, employment, ethnicity, gestational age at booking, maternal age at delivery, parity, year of delivery and region are shown in Table 1. There was a fairly even distribution of women across the IMD quintiles, the majority of women were White (84.9%), employed (65.0%), booked within the first trimester (59.4%), aged 25–29 (28.2%) or 30.34 (26.7%), had a parity of 0 (36.6%) or 1 (35.6%). Although data were available from 1990 to 2007 and for eight regions in England, the majority of included data were for births post 2002 (74.1%) and women from the South East of England were represented more than other regions (39.3%) (Table 1).

**Table 1:**
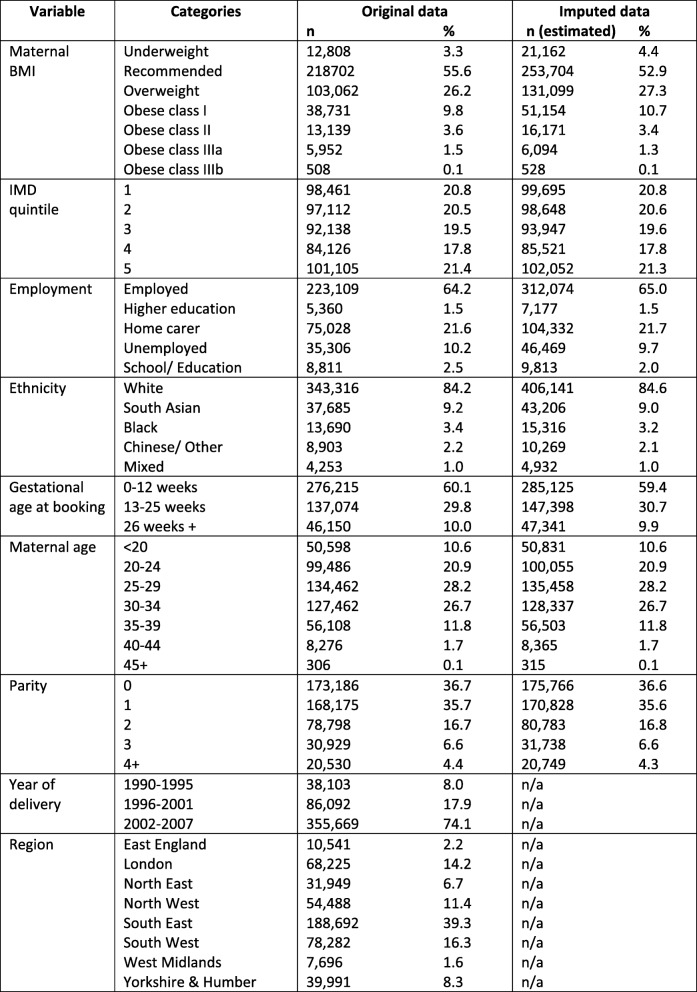
Study population socio-demographics, imputed and original data

Footnote: Year of delivery and region were complete and therefore multiple imputation was not required.

Abbreviations: *BMI* Body mass index, *IMD* Index of multiple deprivation, *n/a* Not applicable

### Preterm birth

There was a J-shaped association between the absolute risk of extreme preterm birth and maternal BMI category (0.8, 0.6, 0.6, 0.7, 0.8, 0.8, and 1.4% for underweight, recommended weight, overweight and obese class: I, II, IIIa, IIIb respectively, Table 2). Compared with women in the recommended weight category, the AORs for extreme preterm birth were significantly increased for all obesity classes, with increasing effect size with increasing obesity class, most notably for obesity class IIIb (class I: 1.20 (95% CI 1.03–1.40), class II 1.39 (95% CI 1.13–1.71), class IIIa 1.52 (95% CI 14.14–2.03), and class III3b 2.80 (95% CI 1.31–5.98), Table 3). When combining all classes of maternal obesity into one obesity category, the AOR for extreme preterm birth was 1.33 (95% CI 1.17–1.52, Table 4); higher than the AOR for class I obesity but lower than the AORs for classes II, IIIa and IIIb. The adjusted odds of extreme preterm birth were also significantly increased for maternal underweight and overweight categories (Table 3). The sensitivity analysis for extreme preterm birth when setting a higher gestational age limit to reflect the accepted limit of viability (24–27 weeks) identified an increased association with underweight, class II, IIIa and IIIb obesity compared with the AORs for extreme preterm birth when using the lower gestational age limit [20–27], and a decreased association for overweight and class I obesity (Table 3).

**Table 2:**
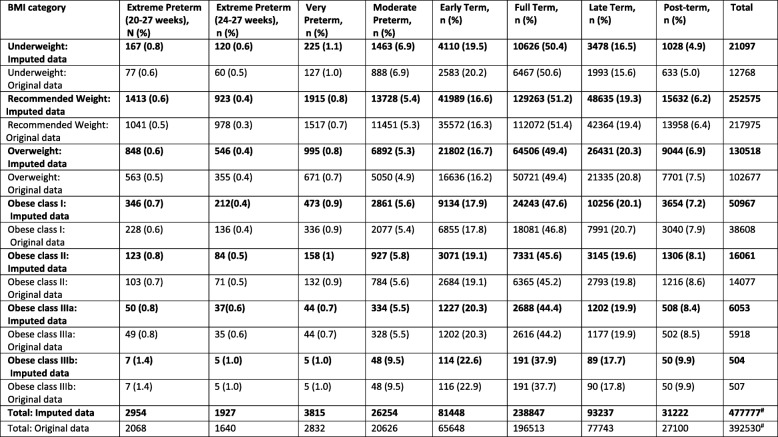
Estimated absolute risk of gestational age at delivery categories according to BMI category using original (non-imputed) and imputed data

# 2087 (0.4%) with missing gestational age were excluded. Total including extreme preterm births 20-27 weeks gestation. NOTE: Frequencies for the imputed data were estimated from the imputed percentages, and therefore may not sum exactly due to rounding errors

**Table 3:**
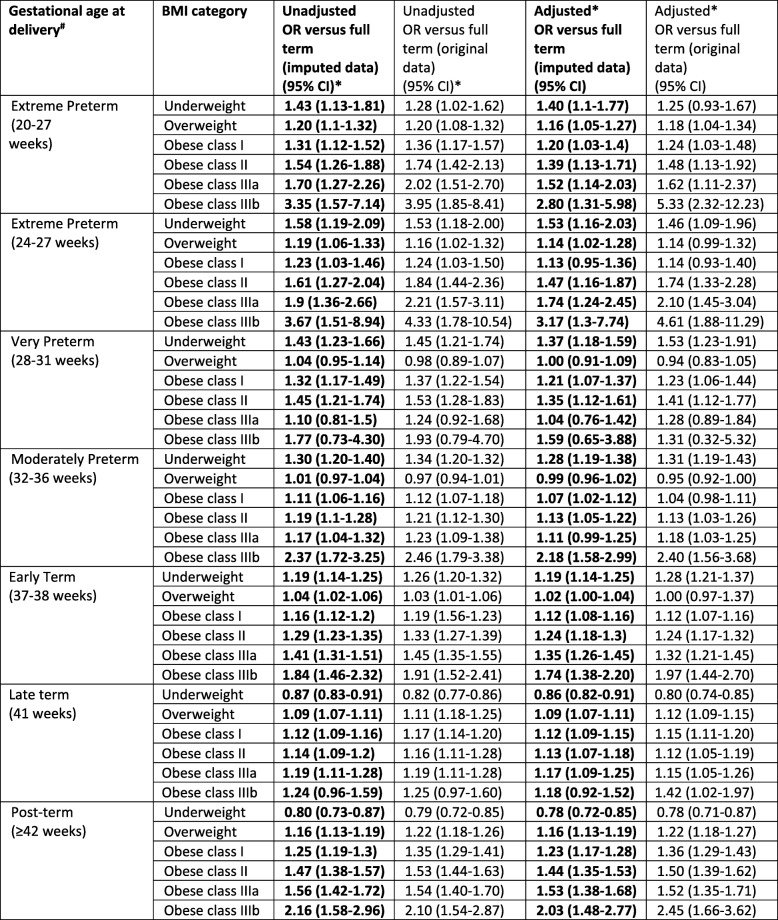
Unadjusted and adjusted odd ratios representing the associations between gestational age at delivery and maternal BMI categories

# Full term birth (39-40 weeks) was the reference group for all gestational age comparisons

*Recommended BMI was the reference group for all BMI comparisons

¥ Adjusted for BMI category, IMD quintile, ethnicity, employment, maternal age, parity, gestational age at booking and year of deliver

**Table 4:**
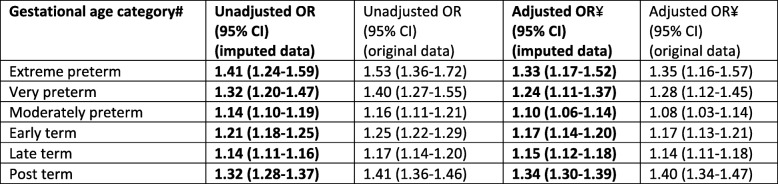
Unadjusted and adjusted odd ratios (estimated using imputed data) representing the associations between gestational age at delivery and combined maternal obesity classes (BMI ≥30kgm2)*

# Full term birth was the reference group for all gestational age comparisons

*Recommended BMI was the reference group for all BMI comparisons

¥ Adjusted for BMI category, IMD quintile, ethnicity, employment, maternal age, parity, gestational age at booking and year of delivery

A J-shaped association was also observed between maternal BMI and very and moderate preterm birth (Table 2). The AORs for very preterm were significantly increased for maternal obesity classes I (1.21, 95% CI 1.07–1.37) and II (1.35, 95% CI 1.12–1.61), and for maternal underweight; however, there was no significant association with obesity classes IIIa/b or with maternal overweight (Table 3). The odds of moderate preterm birth were significantly increased for all obesity classes except IIIa; the greatest effect size was for obesity class IIIb: (1.07, 95% CI 1.02–1.12; 1.13, 95% CI 1.05–1.22; and 2.18, 95% CI 1.58–2.99 for classes I, II and IIIb respectively). There was also a significantly increased AOR for maternal underweight but not for maternal overweight (Table 3). When combining all maternal obesity categories, a similar pattern was observed for both very and moderate preterm birth with the AOR for overall obesity falling between the AORs for class I and II obesity (Table 4); therefore overestimating the risk for women with class I obesity and underestimating for the other obesity classes (although not all had significant associations for these two outcomes).

### Post-term birth

The absolute risk of post-term birth increased monotonically as BMI category increased (4.9, 6.2, 6.9, 7.2, 8.1, 8.4, and 9.9% for underweight, recommended, overweight and obese class: I, II, IIIa and IIIb respectively, Table 2). Compared to women in the recommended weight category, the AORs were significantly increased for all obesity classes with the effect sizes increasing almost linearly with increasing maternal BMI category, although there was a steeper increase in effect size for women with class IIIb obesity (2.03, 95% CI 1.48–2.77, Table 3). There was also a significantly increased AOR for maternal overweight and significantly reduced AOR for maternal underweight (Table 3). When considering all obese classes combined, the AOR again showed a similar pattern to the analyses of preterm birth. The AOR was 1.34 (95% CI 1.30–1.39, Table 4) which falls in between obesity class I and II (AOR 1.23 (95% CI 1.17–1.28) and AOR 1.44 (95% CI 1.35–1.53) respectively, Table 3).

### Term birth

There was a J-shaped association between maternal BMI and absolute risk of early term birth (Table 2). The pattern of AORs mirrored that observed in the preterm birth categories with a significantly increased association for all obesity classes, most notably class IIIb (1.74, 95% CI 1.38–2.20) but with generally smaller effect sizes (Table 3). There was also a significantly increased AOR for maternal underweight and borderline significance for overweight categories. The odds of late term birth increased monotonically with increasing maternal BMI, mirroring the association observed between post–term birth and maternal BMI, with a significantly reduced AOR for maternal underweight and linear increase in AORs for maternal overweight through to obesity class IIIb (Table 3).

## Discussion

This study aimed to determine the associations between maternal obesity classes and categories of gestational age at birth. To the best of our knowledge, this was the first study to explore the association between categories of gestational age at birth; in particular extreme preterm birth, and maternal extreme obesity. With the exception of very preterm birth, the results showed a significantly increased association with all obesity classes and gestational age categories. There was a consistent J-shaped association for all preterm and early term birth categories, showing an increased risk for both maternal underweight and obesity, most notably for maternal obesity class IIIb. There was also a linear association with post-term and late term birth categories with a significantly reduced association for maternal underweight, and increasing association with obesity classes; again most notably with obesity class IIIb.

The associations identified in this research add evidence to the argument that combining subclasses of maternal obesity does not accurately represent the risks for many women with obesity. Doing so resulted in an overestimation of risk of preterm and post-term birth for women with class I obesity, and an underestimation of risk for women in all other obesity classes. This is especially the case for women with class IIIb obesity. Two previous studies reporting on class IIIb obesity also showed increased risks including pre-eclampsia, gestational diabetes, admission to intensive care and caesarean section, large for gestational age and a 5-min Apgar score < 7 [14, 15]. Risks were increased compared with all other BMI categories, including women with obesity (> 30 to < 50 kg/m^2^). We also found that women with class IIIb obesity were at the greatest risks of both preterm and post-term birth. In particular, this population had almost 3-fold increased odds of extreme preterm birth (20–27 weeks gestation) and more than 3-fold increase when restricting to the gestation for viability (24–27 weeks). There are significant inequalities associated with class IIIb obesity, especially relating to socio-economic status. UK data shows that women with class IIIb obesity have almost five-times increased odds of living in areas of highest deprivation than women with a recommended BMI, following adjustment for additional socio-demographic confounders [13]. Therefore, our findings reflect a double burden of inequality for mothers who face the highest levels of deprivation and the greatest risk of adverse preterm and post-term outcomes, and subsequent inequality for lifelong health of their offspring. For all preterm infants, costs associated with the provision of neonatal intensive care are increased [32]. In addition, the low chance of survival for infants born below the limit of viability [26] raises a number of difficult decisions for both parents and health professionals in relation to resuscitation [33]. Although obesity alone may not be an indication for induction of labour, the association between maternal obesity and post-term birth observed by both this study and other published research suggests an increase in risk of the requirement for either induction of labour or increased antenatal monitoring.

Our study was not able to distinguish between spontaneous and medically indicated preterm births. This is important given that women with obesity are more likely to develop comorbidities such as gestational diabetes or pre-eclampsia, which can necessitate early delivery [34]. A study in the US investigated the risk of spontaneous and indicated preterm birth for women with class I obesity, and combined classes II/III [35]. The authors from the US study identified significantly increased relative risks (RR) for both classes (obesity class I, and combined classes II/III) and both spontaneous and indicated extreme preterm birth (defined as 23–27 weeks) compared to term birth (defined as ≥37 weeks). They reported a significantly increased RR for both obesity classes and indicated moderate/late preterm birth (32–36 weeks) and also for class II/III obesity and indicated very preterm birth (28–31 weeks). However, they found no significant increased risk for either of the obesity classes and spontaneous very or moderate/late preterm birth, or for indicated very preterm birth for class II/III obesity. Our study adds to this evidence by investigating both the sub-classes of obesity and subcategories of term birth.

### Strengths and limitations

This study used national data for England and included a large sample of 479,864 live births from eight regions, which had 99.6% complete gestational age data. The large sample size meant we had the power to investigate important sub-classes of both maternal obesity including extreme obesity, and gestational age including extreme preterm birth. Although we did not do any power calculation for the analysis, previous studies investigating maternal extreme obesity used samples of 665 and 370 women with extreme obesity. Knight et al found that in a sample of 764,387 women in the UK, 665 had extreme obesity (estimated a prevalence of 8.7 per cases of extreme obesity per 10,000 pregnant women) [14]. Sullivan et al identified 370 women with extreme obesity in Australia (from approximately 171,289 women giving birth) [36]. With these sample sizes, both studies were able to consider the association between pregnancy outcomes and extreme obesity. Based on the prevalence estimate for a UK population by Knight et al., [14] in our sample we would expect 418 cases of extreme obesity (the actual number of cases in our study exceeded this: *n* = 508 women).

To our knowledge, this is the first study of this kind. We were able to show that the risk of preterm and post-term birth is not uniform in mothers with a BMI ≥ 30kgm^2^, but increases with increasing obesity classes. Crucially, we were able to analyse the association between maternal obesity class IIIb and extreme preterm birth; this is a novel finding and important to maternal services given the rapid increase in the prevalence of class III obesity over time [13] and the large effect size observed. The sample size was also sufficient to apply the recommended subcategories of term birth, an under-research area in the context of maternal obesity, and showed a varied association. Our results showed that although that there may be little clinical significance in subdividing term birth for women with overweight and obesity, this may be a useful approach for women with an underweight BMI as there was a higher proportion of women in this BMI group delivering early term.

We had access to multiple socio-demographic variables enabling us to control for important confounding factors and reasonably impute missing values under the missing at random assumption. The use of multiple imputation with chained equations, as opposed to complete case analysis, meant we were able to reduce bias in our estimates due to missingness, while also maximising our sample size. However, the dataset did not contain information on other variables, which may be important confounders. For example, we may have introduced unobserved confounding because we were not able to adjust for smoking, which is associated with preterm birth [37] and maternal BMI [38]. Finally, the aim of this study was to investigate both obesity sub-classes and the subcategories of gestational age, resulting in multiple testing of outcomes. Due to random variation, we would expect one in twenty comparisons to be statistically significant at the 5% level and multiple comparisons increase the likelihood that a statistically significant result may be observed [39]. Caution should be taken when interpreting the *p*-values; although the effect sizes show evidence that the odds of pre- and post-term birth increase across maternal BMI categories. Due to the large sample size it is also important to keep in mind that small absolute differences may be statistically significantly for example although women with class IIIb obesity have approximately a 3-fold increase in risk for extreme preterm birth, this outcome is rare, and only 1.4% of all women have an extreme preterm birth.

## Conclusion

There is a J-shaped association between maternal BMI and extreme preterm, very preterm, moderate preterm and early term birth, and a linear association with late term and post-term birth. While few studies have researched class IIIb obesity, we found that this population had the greatest odds of preterm and post–term birth, particularly for extreme preterm birth, which results in the greatest risks for offspring survival and lifelong health. Given the strong association with class IIIb obesity and deprivation, these women and their children are at particular risk of health inequalities and should be a priority for future research, public health and clinical guidelines.

We have added evidence to the need for pregnancy-related research and practice to consider all obesity classes separately; obesity is a heterogeneous population as not all obesity classes have same level of risk or care requirements. Additionally, we have shown that early, full and late term births have very different associations with maternal BMI, where early term reflects the patterns observed among preterm birth, and late term reflects the patterns observed with post-term birth. Term birth sub-categories should therefore be considered separately in line with the 2013 guideline recommendations when estimating risk of gestational age as an outcome, and also based on evidence that new-born outcomes are not uniform after 37 weeks [19].

## Data Availability

Data used in this study are not currently publicly available. Requests for access to the dataset should be made to the corresponding author, and will require all necessary ethical approvals and data sharing agreements to be in place.

## Abbreviations

AOR: Adjusted odds ratio
BMI: Body Mass Index
CI: Confidence interval
IMD: Index of multiple deprivation
NHS: National Health Service
OR: Odds ratio
UK: United Kingdom
USA: United States of America
WHO: World Health Organisation
